# Incorporated Biochar-Based Soil Amendment and Exogenous Glycine Betaine Foliar Application Ameliorate Rice (*Oryza sativa* L.) Tolerance and Resilience to Osmotic Stress

**DOI:** 10.3390/plants10091930

**Published:** 2021-09-16

**Authors:** Emad M. Hafez, Salah M. Gowayed, Yasser Nehela, Raghda M. Sakran, Asmaa M. S. Rady, Abdelmoniem Awadalla, Alaa El-Dein Omara, Bassam F. Alowaiesh

**Affiliations:** 1 Department of Agronomy, Faculty of Agriculture, Kafrelsheikh University, Kafr El-Sheikh 33516, Egypt; emadhafez2012@agr.kfs.edu.eg; 2 Department of Botany, Faculty of Agriculture, Suez Canal University, Ismailia 41522, Egypt; salahgowed@yahoo.com; 3 Department of Agricultural Botany, Faculty of Agriculture, Tanta University, Tanta 31527, Egypt; 4 Citrus Research and Education Center, Department of Plant Pathology, University of Florida, 700 Experiment Station Rd., Lake Alfred, FL 33850, USA; 5 Rice Research Department, Field Crops Research Institute, Agricultural Research Center, Giza 12112, Egypt; raghdasakran@yahoo.co.uk; 6 Crop Science Department, Faculty of Agriculture (EL-Shatby), Alexandria University, Alexandria 21545, Egypt; asmaa.mohamed@alexu.edu.eg; 7 Department of Agronomy, Faculty of Agriculture and Natural Resources, Aswan University, Aswan 81528, Egypt; abdelmonemomr@yahoo.com; 8 Department of Microbiology, Soils, Water and Environment Research Institute, Agricultural Research Center, Giza 12112, Egypt; ala.omara@yahoo.com; 9 Biology Department, College of Science, Jouf University, Sakaka 72341, Saudi Arabia; bfalawish@ju.edu.sa

**Keywords:** rice, biochar, glycine betaine, osmotic stress, soil salinity, water stress, plant resilience

## Abstract

Osmotic stress is a major physiologic dysfunction that alters the water movement across the cell membrane. Soil salinity and water stress are major causal factors of osmotic stress that severely affect agricultural productivity and sustainability. Herein, we suggested and evaluated the impact of integrated biochar-based soil amendment and exogenous glycine betaine application on the growth, physiology, productivity, grain quality, and osmotic stress tolerance of rice (*Oryza sativa* L., cv. Sakha 105) grown in salt-affected soil under three irrigation intervals (6, 9, or 12 days), as well as soil properties and nutrient uptake under field conditions during the 2019 and 2020 seasons. Our findings showed that dual application of biochar and glycine betaine (biochar + glycine betaine) reduced the soil pH, electrical conductivity, and exchangeable sodium percentage. However, it enhanced the K^+^ uptake which increased in the leaves of treated-rice plants. Additionally, biochar and glycine betaine supplementation enhanced the photosynthetic pigments (chlorophyll *a*, *b*, and carotenoids) and physiological attributes (net photosynthetic rate, stomatal conductance, relative water content, and electrolyte leakage) of osmotic-stressed rice plants. Biochar + glycine betaine altered the activity of antioxidant-related enzymes (catalase, ascorbate peroxide, and peroxidase). Moreover, it improved the yield components, biological yield, and harvest index, as well as the nutrient value of rice grains of osmotic-stressed rice plants. Collectively, these findings underline the potential application of biochar and glycine betaine as a sustainable eco-friendly strategy to improve plant resilience, not only rice, but other plant species in general and other cereal crops in particular, to abiotic stress, particularly those growing in salt-affected soil.

## 1. Introduction

Rice (*Oryza sativa* L.) is the main imperative food crop that feeds most people all over the world [1]. In Egypt, rice is grown on over 0.5 million hectares, with a productivity estimated at 6.1 million tons according to the FAO report 2020 [2]. The current and projected global food requires a significant increase in crop productivity in less favorable lands [3]. Climate change poses a major threat to agricultural production, particularly in developing countries, resulting in abiotic stresses such as soil salinity and water stress on plant growth and production especially in the arid and semi-arid ecosystems [4]. It is imperative to minimize the environmental stresses to increase rice yield with improved soil quality [5]. The world population is growing promptly annually, which requires the need to increase the agricultural production in field crops such as rice, on which most of the world’s population depends, especially in Egypt by 2050 [6].

Nevertheless, environmental stressors like water stress and soil salinity seriously menace crop yield and trigger significant yield loss in arid and semi-arid regions. Rice is very susceptible to water deficit through the different growth stages, resulting in significant crop loss owing to abiotic stress [7]. By the end of the twenty-first century, crop yield can reduce by 70% due to water stress compared to the optimal yield. Exposure of plants to drought and water shortage causes a lessening in uptake and transport of nutrients, chloroplast damage, as well as stimulates mature and young leaf senescence [8]. Soil salinity is another severe abiotic stress, which accounts for a serious threat to crop production and is anticipated to increment owing to universal climate changes and as a result of water stress [9]. Roughly 830 Mha of land worldwide are influenced by salinity, around a third of it being in Africa. The negative impacts of soil salinity on crop development are linked with the low osmotic potential of the soil solution, resulting in osmotic, ionic, and oxidative stress, along with nutritional imbalances [10]. To cope with water deficit and soil salinity, there are several strategies, such as breeding of new cultivars and new alternative agricultural practices [11].

Concerning the current global climate change, drought stress became the most imperative and serious limiting factor for rice production in rainfed ecosystems [12]. More than one-third of the total rice-cultivated area is affected by drought stress worldwide [12]. In Asia alone, there are approximately two-thirds of the upland rice area, about 34 million square hectometers (hm^2^) of rainfed lowland, and around eight million hm^2^ of upland rice exposed to drought stress [13,14]. Rice breeding and development of drought-tolerant varieties is an economically viable and sustainable option to improve rice productivity [15]; however, it takes a long time. Another option to partially cope with the drought stress is via refining sustainable agricultural practices for rice. Adjusting the irrigation intervals is a key agricultural practice to grow rice under drought conditions.

Recovering soil quality is an important issue for improving withholds water and nutrients via soil as well as rice growth and productivity particularly under environmental stressors including salt and drought stressors [16]. Soil amendment with biochar can be beneficial and effective for high yield. Biochar is a carbon-rich solid material produced by pyrolyzing biomass (e.g., crop residues) in an oxygen-limited environment [17]. Biochar application to soils is deemed a sustainable organic additive to sequester carbon from the environment, improved moisture-holding capacity, and enhanced soil health and quality by improving cation exchange capacity organic matter status and soil fertility by nutrient retention and promoting microbial activities in soil [18]. It has been well-known that biochar addition can decrease nitrous oxide emission and N leaching from rice fields and may consequently increment N absorption. Biochar addition enhanced plant water relations by lessening soil evaporation and augmented tolerance versus soil salinity and water stress conditions [19]. Biochar had pronounced impacts to increase the grain yield and production [20].

Various management practices including exogenous application of plant growth regulators, bio-stimulative compounds, or osmoprotectants can play a pivotal role in protecting plants from salt- and drought stresses that improve tolerance in plants [21]. Glycine betaine is endogenously produced in plants and is considered a naturally occurring bio-stimulative compound that accumulates in many plants under various environmental stressors [22]. Salt and drought stressors negatively influenced the growth parameters and yield in rice plants, while it was found that exogenous application of glycine betaine was effective in alleviating the harmful impacts of the environmental stressors as a consequence to improve turgor potential, diminish the oxidative impacts of ROS, maintain the osmotic regulation, improve the K^+^ accumulation, reduce Na^+^, and improve the activity of antioxidant enzymes, therefore improving photosynthesis and reducing oxidative damage [23]. Additionally, glycine betaine can improve growth and production [24]. However, no investigation has been carried out to appraise the relative impact of glycine betaine in improving rice tolerance against salt and drought stresses. Thus, it is very imperative to increase cost-effective strategies to alleviate the harmful impacts of salinity and drought stressors on rice plants.

There are no reports about the dual application of biochar and glycine betaine, especially under soil salinity and water stress conditions together in the field. To contribute to this goal, in this study, we are supposed to determine the dual application of biochar and glycine betaine for mitigating soil salinity and water stress by using rice as a test plant. It is expected that the research findings will be effective for formulating novel management strategies for enhancing growth and rice productivity under soil salinity and water stress under field conditions. Moreover, the findings of this study might be beneficial to grow rice drought-affected areas, as well as upland rice areas worldwide.

## 2. Results

### 2.1. Soil Amendment Using Biochar and Glycine Betaine Application Altered the Soil Physicochemical Properties

**Figure 1:**
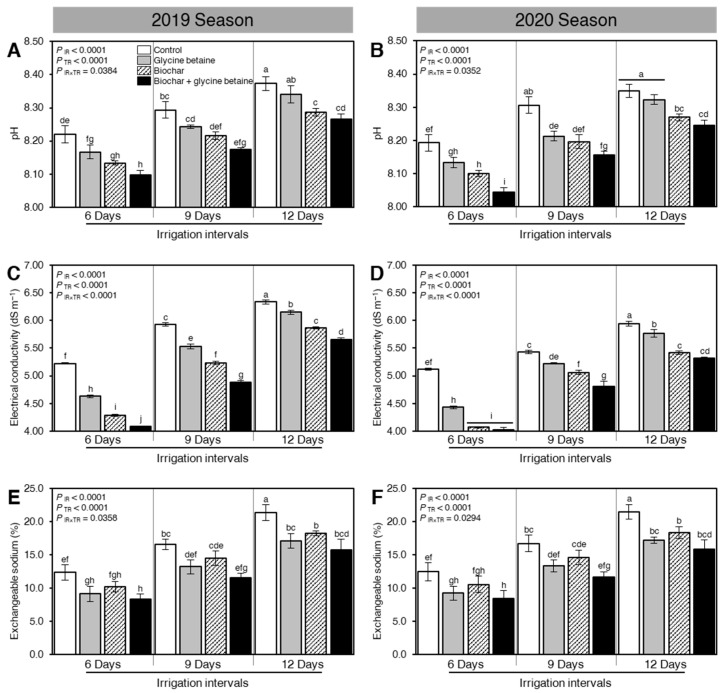
Effect of biochar-based soil amendment and exogenous glycine betaine application on the physicochemical properties of soil planted with rice at maturity stage under three irrigation intervals (6, 9, and 12 days). (**A**,**B**) soil pH, (**C**,**D**) electrical conductivity (EC; dS m^−1^), and (**E**,**F**) exchangeable sodium percentage (ESP) during two successive seasons, 2019 and 2020, respectively. Bars and error bars represent the means and SDs, respectively, of three biological replicates. Different letters indicate statistically significant differences among treatments (Tukey HSD; *P*_IR×TR_ < 0.05).

The effect of exogenous application of biochar and glycine betaine on the soil physicochemical properties including pH, electrical conductivity (EC), and exchangeable sodium percentage (ESP) were studied in two separate field trials (Figure 1). Although the irrigation intervals significantly increased the three studied soil properties (pH, EC, and ESP; *P*_IR_ < 0.0001) in both seasons, soil amendment using biochar and/or glycine betaine application significantly decreased all studied properties at all tested irrigation intervals (*P*_TR_ < 0.0001) (Figure 1). Briefly, in 2019, the dual application of biochar and glycine betaine significantly decreased the soil pH when rice plants were watered every 6, 9, or 12 days (8.1 ± 0.02, 8.17 ± 0.01, and 8.27 ± 0.02, respectively; *P*_IR×TR_ = 0.0384) compared with non-treated control (8.22 ± 0.03, 8.29 ± 0.03, 8.37 ± 0.02, respectively) (Figure 1A). Interestingly, the same pH profile was noticed during the 2020 season (Figure 1B; *P*_IR×TR_ = 0.0352).

Likewise, exogenous application of biochar, glycine betaine, or both together significantly reduced the soil EC during 2019 (Figure 1C) and 2020 (Figure 1D). The combined application of both biochar and glycine betaine had the lowest EC values at all three studied irrigation intervals (6, 9, or 12 days) during the 2019 (4.08 ± 0.01, 4.88 ± 0.03, and 5.66 ± 0.03 dS m^−1^, respectively; Figure 1C) and 2020 (4.02 ± 0.04, 4.81 ± 0.09, and 5.32 ± 0.02 dS m^−1^; Figure 1D) seasons compared with control in the 2019 (5.22 ± 0.02, 5.93 ± 0.03, and 6.34 ± 0.04 dS m^−1^, respectively) and 2020 seasons (5.12 ± 0.02, 5.43 ± 0.03, and 5.94 ± 0.05 dS m^−1^, respectively). Similarly, in 2019, ESP was significantly reduced due to the dual application of biochar and glycine betaine together when rice plants watered every 6, 9, or 12 days (8.32 ± 0.82, 11.57 ± 0.64, and 15.75 ± 1.56%, respectively; *P*_IR×TR_ = 0.0358) compared with control (12.36 ± 1.13, 16.58 ± 0.79, and 21.36 ± 1.17% respectively; Figure 1E). It is worth mentioning that a typical ESP profile was noticed when the whole experiment was repeated during the 2020 season (Figure 1F).

### 2.2. Exogenous Application of Biochar and Glycine Betaine Modified the Leaf Na^+^ and K^+^ Contents at the Anthesis Stage of Stressed Rice Plants

**Figure 2:**
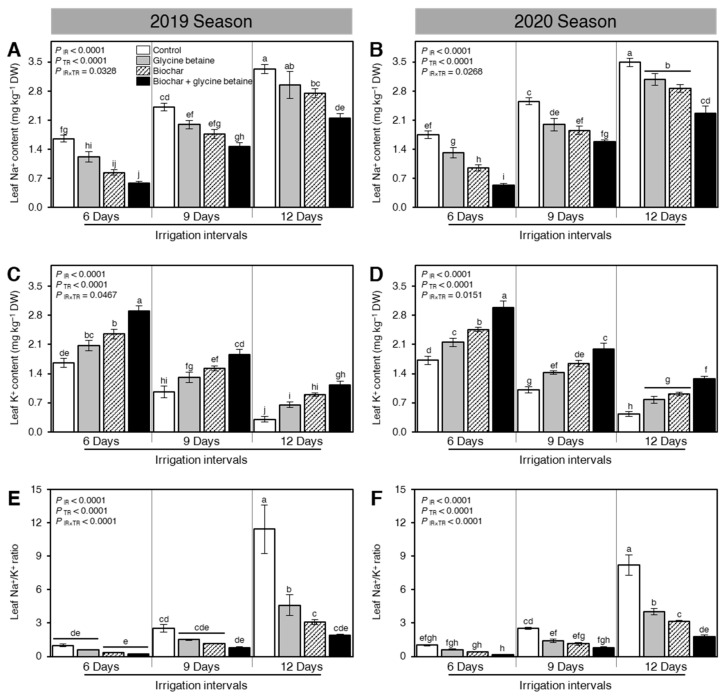
Effect of biochar-based soil amendment and exogenous glycine betaine application on the leaf Na^+^ and K^+^ contents at the anthesis stage of osmotic-stressed rice plants under three irrigation intervals (6, 9, and 12 days). (**A**,**B**) Leaf Na^+^ content (mg kg^−1^ DW), (**C**,**D**) Leaf K^+^ content (mg kg^−1^ DW), and (**E**,**F**) Leaf Na^+^/K^+^ ratio during two successive seasons, 2019 and 2020, respectively. Bars and error bars represent the means and SDs, respectively, of three biological replicates. Different letters indicate statistically significant differences among treatments (Tukey HSD; *P*_IR×TR_ < 0.05).

Although increasing the irrigation intervals, from 6 days to 9 or 12 days, significantly boosted the leaf Na^+^ content (mg kg^−1^ DW; *P*_IR_ < 0.0001, Figure 2A,B), it dramatically reduced the K^+^ content (mg kg^−1^ DW; *P*_IR_ < 0.0001, Figure 2C,D) of osmotic stressed rice plants in both seasons 2019 and 2020. Moreover, soil amendment using biochar and/or glycine betaine application considerably lessened the leaf Na^+^ content at all tested irrigation intervals (*P*_TR_ < 0.0001; Figure 2A,B). On the other hand, the dual exogenous application of biochar and glycine betaine together significantly enhanced the K^+^ content of rice leaves (*P*_TR_ < 0.0001, Figure 2C,D) at all tested irrigation intervals. It is worth mentioning that the alteration in the leaf Na^+^ and K^+^ contents significantly affected the leaf Na^+^/K^+^ ratio during 2019 and 2020 (Figure 2E,F, respectively). Briefly, the lowest Na^+^/K^+^ ratios of all tested treatments were recorded when rice plants were irrigated every 6 days; however, increasing the irrigation intervals to 9 or 12 days significantly increased the Na^+^/K^+^ ratios in all treatments during both seasons. In both seasons, the highest leaf Na^+^/K^+^ ratio was noticed from the control plants that were watered every 12 days (Figure 2E,F, respectively).

### 2.3. Biochar and Glycine Betaine Supplementation Altered the Proline Content of Rice Leaves

**Figure 3:**
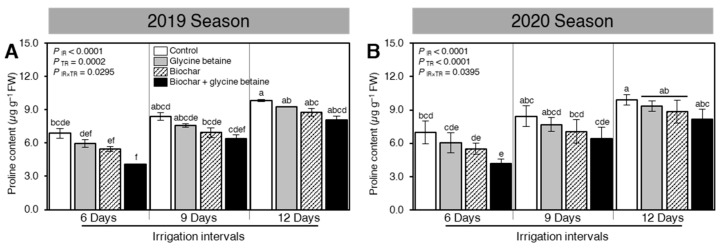
Effect of biochar-based soil amendment and exogenous glycine betaine application on the endogenous proline content of osmotic-stressed rice plants under three irrigation intervals (6, 9, and 12 days). (**A**,**B**) Leaf proline content (µg g^−1^ FW) during the 2019 and 2020 seasons, respectively. Bars and error bars represent the means and SDs, respectively, of three biological replicates. Different letters indicate statistically significant differences among treatments (Tukey HSD; *P*_IR×TR_ < 0.05).

The effect of exogenous application of biochar and/or glycine betaine on the endogenous content of the stress-associated amino acid proline was determined (Figure 3). Briefly, increasing the irrigation intervals, from 6 days to 9 or 12 days, considerably improved the proline content during both seasons 2019 and 2020 (*P*_IR_ < 0.0001, Figure 3A,B, respectively). Regardless of the biochar and glycine betaine treatments, the highest proline levels were recorded when rice plants were irrigated every 12 days. Nevertheless, in both seasons, integrated biochar-based soil amendment and glycine betaine supplementation lowered the proline accumulation in treated plants compared with the control ones.

### 2.4. Soil Amendment Using Biochar and Exogenous Glycine Betaine Supplementation Enhanced the Photosynthetic Pigments

**Figure 4:**
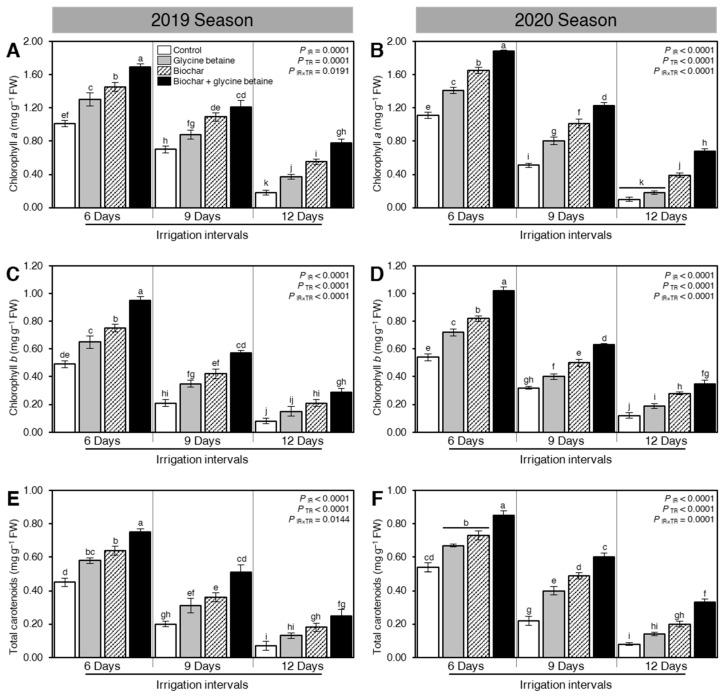
Effect of biochar-based soil amendment and exogenous glycine betaine application on the content of the photosynthetic pigment of osmotic-stressed rice plants under three irrigation intervals (6, 9, and 12 days). (**A**,**B**) chlorophyll *a* (mg g^−1^ FW), (**C**,**D**) chlorophyll *b* (mg g^−1^ FW), and (**E**,**F**) total carotenoids (mg g^−1^ FW) during two successive seasons 2019 and 2020, respectively. Bars and error bars represent the means and SDs, respectively, of three biological replicates. Different letters indicate statistically significant differences among treatments (Tukey HSD; *P*_IR×TR_ < 0.05).

Although osmotic stress reduced the levels of photosynthetic pigments when the irrigation intervals increased from 6 days to 9 or 12 days, soil amendment using biochar and exogenous glycine betaine supplementation enhanced the levels of chlorophyll *a* (Figure 4A,B), chlorophyll *b* (Figure 4C,D), and total carotenoids (Figure 4E,F) during the 2019 and 2020 seasons. In control plants, increasing the irrigation intervals from 6 days to 9 or 12 days significantly reduced the levels of chlorophyll *a* (Figure 4A,B), chlorophyll *b* (Figure 4C,D), and total carotenoids (Figure 4E,F) during the 2019 and 2020 seasons. Generally, rice plants had the highest levels of photosynthetic pigments when irrigated every six days. At 6 day irrigation interval, the dual application of biochar and glycine betaine together had the highest levels of chlorophyll *a* (1.69 ± 0.04 and 1.88 ± 0.02 mg g^−1^ FW), chlorophyll *b* (0.95 ± 0.03 and 1.02 ± 0.03 mg g^−1^ FW), and total carotenoids (0.75 ± 0.02 and 0.85 ± 0.03 mg g^−1^ FW) during the 2019 and 2020 seasons, respectively, followed by biochar alone, then glycine betaine. Although all photosynthetic pigments were significantly reduced at 12 days irrigation intervals, the dual application of biochar and glycine betaine had the highest levels of chlorophyll *a* (0.78 ± 0.05 and 0.68 ± 0.03 mg g^−1^ FW), chlorophyll *b* (0.29 ± 0.03 and 0.35 ± 0.03 mg g^−1^ FW), and total carotenoids (0.25 ± 0.04 and 0.33 ± 0.02 mg g^−1^ FW) during the 2019 and 2020 seasons, respectively.

### 2.5. Exogenous Application of Biochar and Glycine Betaine Improved the Photosynthetic and Physiological Attributes of Osmotic-Stressed Rice Plants

#### 2.5.1. Net Photosynthetic Rate (P_n_)

**Figure 5:**
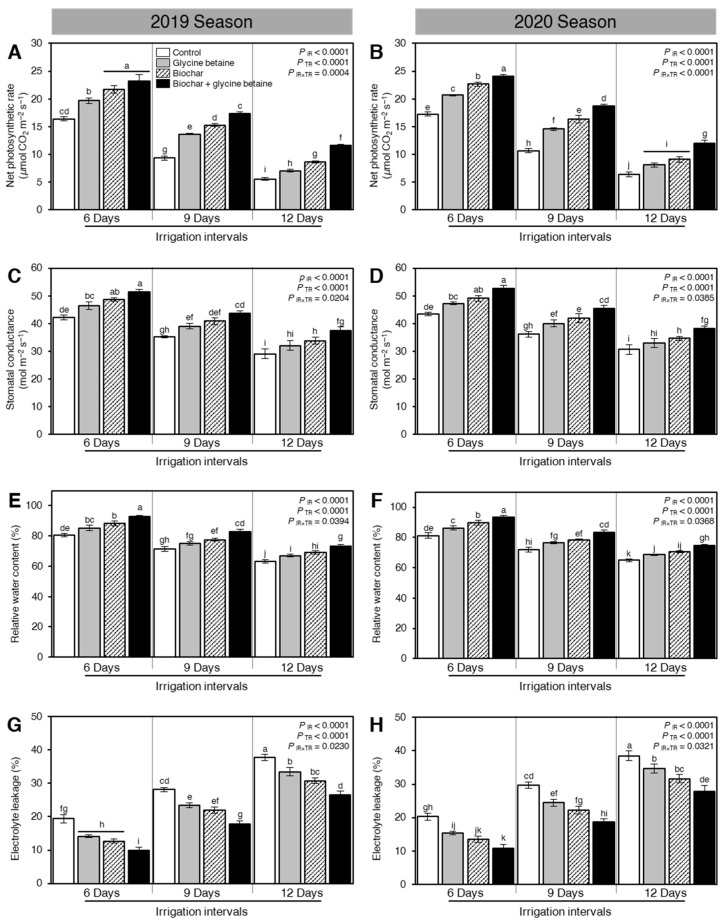
Effect of biochar-based soil amendment and exogenous glycine betaine application on the photosynthetic and physiological attributes of osmotic-stressed rice plants under three irrigation intervals (6, 9, and 12 days). (**A**,**B**) Net photosynthetic rate (μmol CO_2_ m^−2^ s^−1^), (**C**,**D**) Stomatal conductance (mol m^−2^ s^−1^), (**E**,**F**) Relative water content (%), and (**G**,**H**) Electrolyte leakage (%) during two successive seasons, 2019 and 2020, respectively. Bars and error bars represent the means and SDs, respectively, of three biological replicates. Different letters indicate statistically significant differences among treatments (Tukey HSD; *P*_IR×TR_ < 0.05).

In general, during both seasons, the net photosynthetic rate (P_n_) of osmotic-stressed rice plants was diminished when the irrigation intervals increased from 6 days to 9 or 12 days (*P*_IR_ < 0.0001, Figure 5A,B). However, exogenous application of biochar and/or glycine betaine significantly increased the net photosynthetic rate (*P*_IR_ < 0.0001) during the 2019 and 2020 seasons (Figure 5A,B, respectively). At 6 days irrigation interval, the dual application of biochar and glycine betaine had the highest net photosynthetic rates (23.14 ± 1.24 and 24.04 ± 0.35 μmol CO_2_ m^−2^ s^−1^) compared with control plants (16.36 ± 0.38 and 17.25 ± 0.36 μmol CO_2_ m^−2^ s^−1^) during 2019 and 2020, respectively.

#### 2.5.2. Stomatal Conductance (g_s_)

Similar to the net photosynthetic rate, stomatal conductance (g_s_) was significantly reduced when the irrigation intervals increased from 6 days to 9 or 12 days (*P*_IR_ < 0.0001, Figure 5C,D). Nevertheless, soil amendment using biochar and exogenous glycine betaine supplementation enhanced the stomatal conductance at the heading stage of osmotic-stressed rice plants. When rice plants were irrigated every 6 days, the combined application (biochar + glycine betaine) had the highest stomatal conductance (51.36 ± 0.99 and 52.59 ± 1.27 mol m^−2^ s^−1^), followed by biochar alone (48.69 ± 0.66 and 49.14 ± 1.03 mol m^−2^ s^−1^), and glycine betaine alone (46.47 ± 1.34 and 47.25 ± 0.51 mol m^−2^ s^−1^), whereas the control plants had the lowest stomatal conductance (42.25 ± 0.82 and 43.36 ± 0.61 mol m^−2^ s^−1^) during 2019 and 2020, respectively (Figure 5C,D).

#### 2.5.3. Relative Water Content (RWC)

The relative water content of rice leaves was decreased when the irrigation intervals increased from 6 days to 9 or 12 days (*P*_IR_ < 0.0001, Figure 5E,F). Nevertheless, exogenous application of biochar and glycine betaine improved the relative water content of rice leaves at the three tested irrigation intervals (*P*_TR_ < 0.0001). The dual application of biochar and glycine betaine together enhanced the relative water content of rice leaves at 6 (92.98 ± 0.77 and 93.25 ± 1.23%), 9 (82.85 ± 1.65 and 83.45 ± 1.28) and 12 days irrigation intervals (73.44 ± 1.07 and 74.66 ± 0.86%) during the 2019 and 2020 seasons, respectively.

#### 2.5.4. Electrolyte Leakage (EL)

In contrast with relative water content, electrolyte leakage of fully expanded flag leaves of osmotic-stressed rice plants at the flowering stage was increased when the irrigation intervals increased from 6 days to 9 or 12 days (*P*_IR_ < 0.0001, Figure 5G,H). Additionally, integrated biochar-based soil amendment and glycine betaine application significantly reduced the electrolyte leakage of rice flag leaves at all studied irrigation intervals during both seasons. It is worth mentioning that there were no significant differences between biochar and glycine betaine treatments at 6 days irrigation intervals during the 2019 season (Figure 5G).

### 2.6. Biochar and Glycine Betaine Supplementation Altered the Antioxidant-Related Enzymatic Activity in Osmotic-Stressed Rice Plants

**Figure 6:**
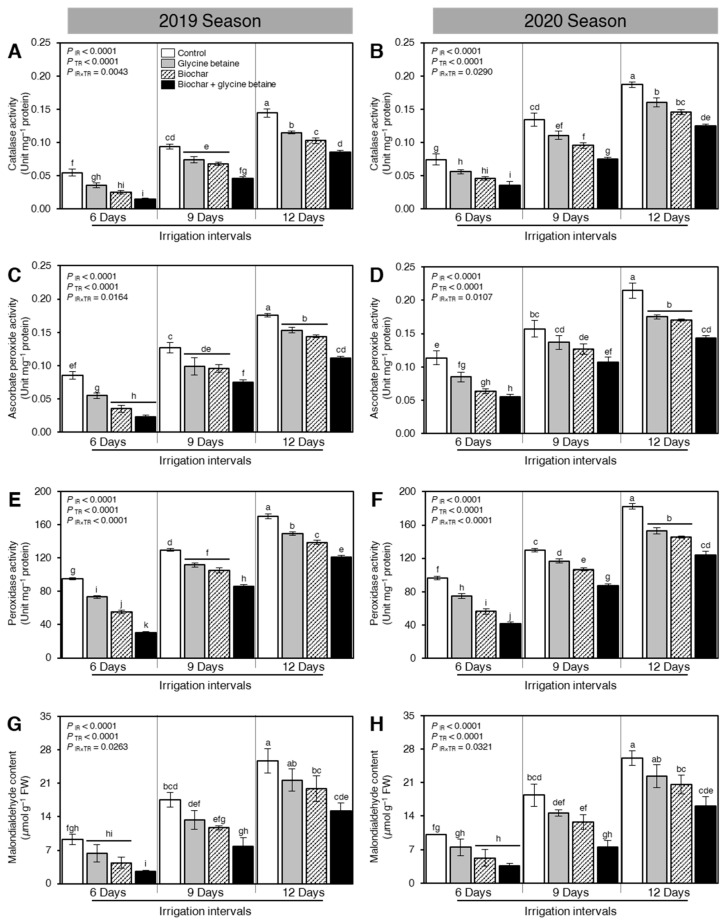
Effect of biochar-based soil amendment and exogenous glycine betaine application on the enzymatic activities of antioxidant-related enzymes of osmotic-stressed rice plants under three irrigation intervals (6, 9, and 12 days). (**A**,**B**) Catalase activity (CAT; Unit mg^−1^ protein), (**C**,**D**) Ascorbate peroxide activity (APX; Unit mg^−1^ protein), (**E**,**F**) Peroxidase activity (POX; Unit mg^−1^ protein), and (**G**,**H**) Malondialdehyde content (MDA; μmol g^−1^ FW) during two successive seasons, 2019 and 2020, respectively. Bars and error bars represent the means and SDs, respectively, of three biological replicates. Different letters indicate statistically significant differences among treatments (Tukey HSD; *P*_IR×TR_ < 0.05).

To better understand how Biochar and glycine betaine supplementation alleviates the oxidative stress in osmotic-stressed rice plants, the enzymatic activities of three antioxidant enzymes including catalase (CAT; Figure 6A,B), ascorbate peroxide (APX; Figure 6C,D), and peroxidase (POX; Figure 6E,F) have been colorimetrically determined during the 2019 and 2020 seasons, respectively. Moreover, the malondialdehyde (MDA; Figure 6G,H) content was also determined as a marker for oxidative stress. Interestingly, the enzymatic activities of all antioxidant-related enzymes (CAT, APX, and POX) and MDA had the same profile during both seasons 2019 and 2020.

Although osmotic stress significantly increased the enzymatic activities of all antioxidant-related enzymes and MDA content when the irrigation intervals increased from 6 days to 9 or 12 days (*P*_IR_ < 0.0001), soil amendment using biochar and exogenous glycine betaine supplementation reduced the activity of CAT (Figure 6A,B), APX (Figure 6C,D), POX (Figure 6E,F), and MDA content (Figure 6A,B) during the 2019 and 2020 seasons. It is worth mentioning that there were no significant differences in the enzymatic activities of the three tested antioxidant enzymes (CAT, APX, and POX) between biochar and glycine betaine treatments at 9 days irrigation intervals during the 2019 season (Figure 6A,C,E, respectively). Likewise, there were no significant differences in the enzymatic activities of APX and POX between biochar and glycine betaine treatments when rice plants were irrigated every 12 days during the 2020 season (Figure 6D,F, respectively).

### 2.7. Integrated Biochar-Based Soil Amendment and Glycine Betaine Application Improved the Yield Components, Biological Yield, and Harvest Index of Stressed Rice Plants

**Figure 7:**
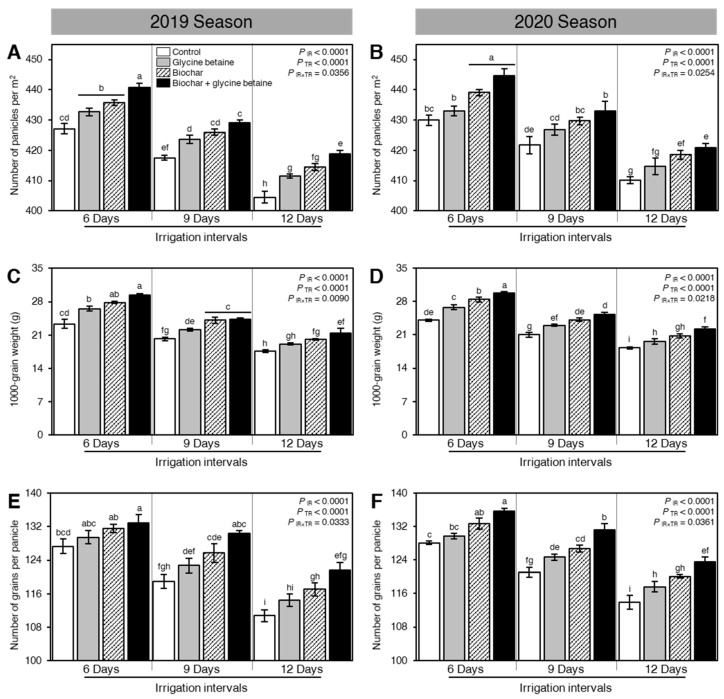
Effect of biochar-based soil amendment and exogenous glycine betaine application on the yield components of osmotic-stressed rice plants under three irrigation intervals (6, 9, and 12 days). (**A**,**B**) Number of panicles per m^2^, (**C**,**D**) 1000-grain weight (g), and (**E**,**F**) Number of grains per panicle during two successive seasons, 2019 and 2020, respectively. Bars and error bars represent the means and SDs, respectively, of three biological replicates. Different letters indicate statistically significant differences among treatments (Tukey HSD; *P*_IR×TR_ < 0.05).

**Figure 8:**
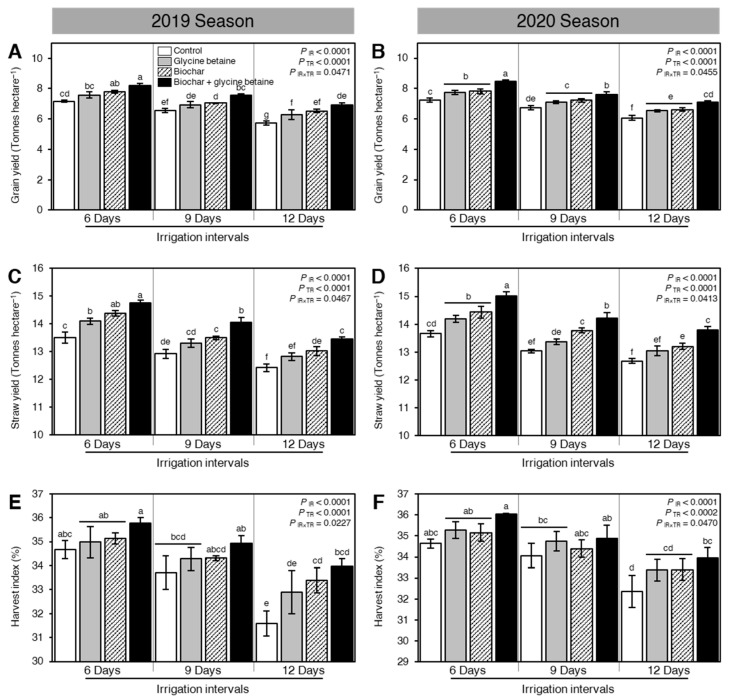
Effect of biochar-based soil amendment and exogenous glycine betaine application on the biological yield and harvest index of osmotic-stressed rice plants under three irrigation intervals (6, 9, and 12 days). (**A**,**B**) Grain yield (Tonnes/hectare), (**C**,**D**) Straw yield (Tonnes/hectare), and (**E**,**F**) Harvest index (%) during two successive seasons, 2019 and 2020, respectively. Bars and error bars represent the means and SDs, respectively, of three biological replicates. Different letters indicate statistically significant differences among treatments (Tukey HSD; *P*_IR×TR_ < 0.05).

Generally, osmotic stress negatively affected the yield and its components including the number of panicles per m^2^ (Figure 7A,B), 1000-grain weight (g; Figure 7C,D), number of grains per panicle (Figure 7E,F), grain yield (tonnes/hectare; Figure 8A,B), straw yield (tonnes/hectare; Figure 8C,D), and harvest index (%; Figure 8E,F). Nevertheless, soil amendment using biochar and exogenous glycine betaine supplementation significantly increased the grain yield and its components. The dual application of biochar and glycine betaine together had the highest number of panicles per m^2^ (440.82 ± 1.25 and 444.70 ± 2.21 panicles m^−2^), 1000-grain weight (29.33 ± 0.40 and 29.76 ± 0.32 g), number of grains per panicle (132.92 ± 1.98 and 135.61 ± 0.81 grains per panicle), grain yield (8.22 ± 0.12 and 8.46 ± 0.10 tonnes/hectare), straw yield (14.76 ± 0.09 and 15.01 ± 0.15 tonnes/hectare), and harvest index (35.76 ± 0.23 and 36.04 ± 0.04%) when the rice plants were irrigated every 6 days during both seasons 2019 and 2020, respectively (Figure 7 and Figure 8). On the other hand, the non-treated, 12-days irrigated, control rice plants had the lowest yield components (number of panicles per m^2^, 1000-grain weight, and number of grains per panicle), biological yield (grain and straw yield), and harvest index.

### 2.8. Biochar and Glycine Betaine Supplementation Enhanced the Nutrient Value of Rice Grains of Osmotic-Stressed Rice Plants

**Figure 9:**
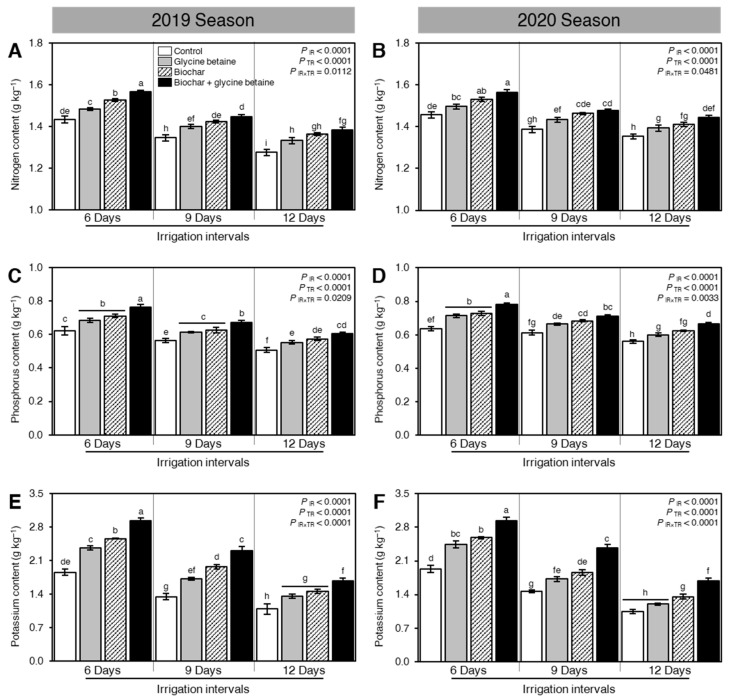
Effect of biochar-based soil amendment and exogenous glycine betaine application on the nutrient value of rice grains of osmotic-stressed rice plants under three irrigation intervals (6, 9, and 12 days). (**A**,**B**) Nitrogen content (g kg^−1^), (**C**,**D**) Phosphorus content (g kg^−1^), and (**E**,**F**) Potassium content (g kg^−1^) during two successive seasons, 2019 and 2020, respectively. Bars and error bars represent the means and SDs, respectively, of three biological replicates. Different letters indicate statistically significant differences among treatments (Tukey HSD; *P*_IR×TR_ < 0.05).

Although osmotic stress reduced the nutrient value of rice grains, as expressed by N, P, and K contents, when the irrigation intervals increased from 6 days to 9 or 12 days (*P*_IR_ < 0.0001), soil amendment using biochar and exogenous glycine betaine supplementation enhanced the nitrogen (Figure 9A,B), phosphorus (Figure 9C,D) and potassium (Figure 9E,F) contents of rice grains during the 2019 and 2020 seasons (*P*_TR_ < 0.0001). Our findings showed that rice grains had the highest N (1.57 ± 0.01 and 1.56 ± 0.02 g kg^−1^), P (0.76 ± 0.02 and 0.78 ± 0.01 g kg^−1^), and K contents (2.93 ± 0.06 and 2.94 ± 0.06 g kg^−1^) during both seasons 2019 and 2020, respectively, when rice plants were treated with biochar and glycine betaine together and irrigated every 6 days. On the other hand, rice grains harvested from control rice plants had the lowest N (1.28 ± 0.02 and 1.35 ± 0.01 g kg^−1^), P (0.51 ± 0.02 and 0.56 ± 0.01 g kg^−1^), and K contents (1.09 ± 0.11 and 1.05 ± 0.04 g kg^−1^) during both seasons 2019 and 2020, respectively, when irrigated every 12 days (Figure 9).

### 2.9. Principal Component Analysis (PCA) Showed a Clear Separation among Treatments

**Figure 10:**
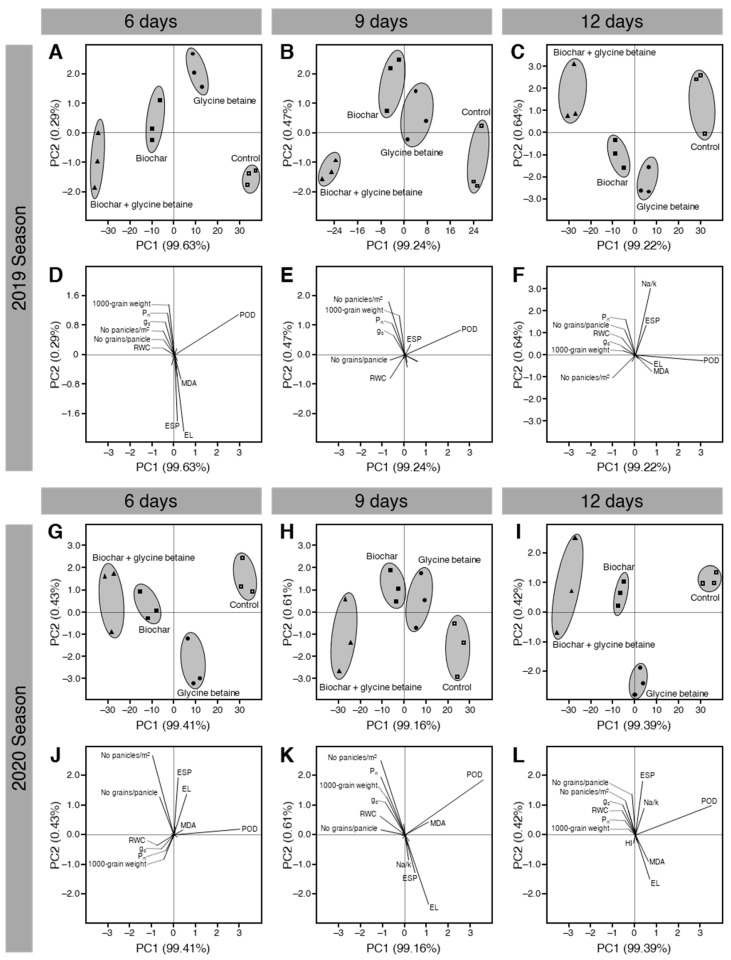
Principal component analysis (PCA) of individual response variables assessed in osmotic-stressed rice plants under three irrigation intervals (6, 9, and 12 days) during the 2019 and 2020 seasons. (**A**–**C**) PCA-associated scatters plots during 2019 at 6, 9, and 12 days irrigation intervals, respectively, (**D**–**F**) PCA-associated loading plots during 2019 at 6, 9, and 12 days irrigation intervals, (**G**–**I**) PCA-associated scatters plots during 2020 at 6, 9, and 12 days irrigation intervals, respectively, and (**J**–**L**) PCA-associated loading plots during 2020 at 6, 9, and 12 days irrigation intervals.

To better understand our data and to extract linear composites of observed variables, principal component analysis (PCA) was carried out (Figure 10). The PCA-associated scatter plot showed a clear separation among all studied treatments (control, glycine betaine-treated, biochar-treated, and biochar + glycine betaine-treated) with respect to PC1 (99.63, 99.24, and 99.22%) and PC2 (0.29, 0.47, and 0.64%) when rice plants irrigated every 6, 9, or 12 days during the 2019 season (Figure 10A–C). Interestingly, the data matrix of non-treated control was clustered separately at the right side of the scatter plot, dual-treated (biochar + glycine betaine) was clustered separately at the left side of the scatter plot, whereas glycine betaine-treated, biochar-treated were clustered together in the center of the scatter plot and separately from other treatments at all tested irrigation intervals 6, 9, and 12 days (Figure 10A–C, respectively). Furthermore, the PCA-associated loading plot showed that while POD enzymatic activity positively correlated with the control treatment, yield components (number of panicles per m^2^, 1000-grain weight, and number of grains per panicle) and photosynthetic attributes (net photosynthetic rate and stomatal conductance) were positively correlated with the dual application of biochar and glycine betaine together at all tested irrigation intervals of 6, 9, and 12 days (Figure 10D–F, respectively). It is worth mentioning that almost the same results were obtained during the 2020 season. The PCA-associated scatter plot showed a clear separation among all studied treatments with respect to PC1 (99.41, 99.16, and 99.39%) and PC2 (0.43, 0.61, and 0.42%) when rice plants were irrigated every 6, 9, or 12 days (Figure 10G–I, respectively) during the 2020 season. Additionally, the PCA-associated loading plot showed that yield components and photosynthetic attributes were positively correlated with the dual application of biochar and glycine betaine together at all tested irrigation intervals of 6, 9, and 12 days (Figure 10J–L, respectively).

### 2.10. Two-Way Hierarchical Cluster Analysis (HCA) Revealed the Differences between Treatments

**Figure 11:**
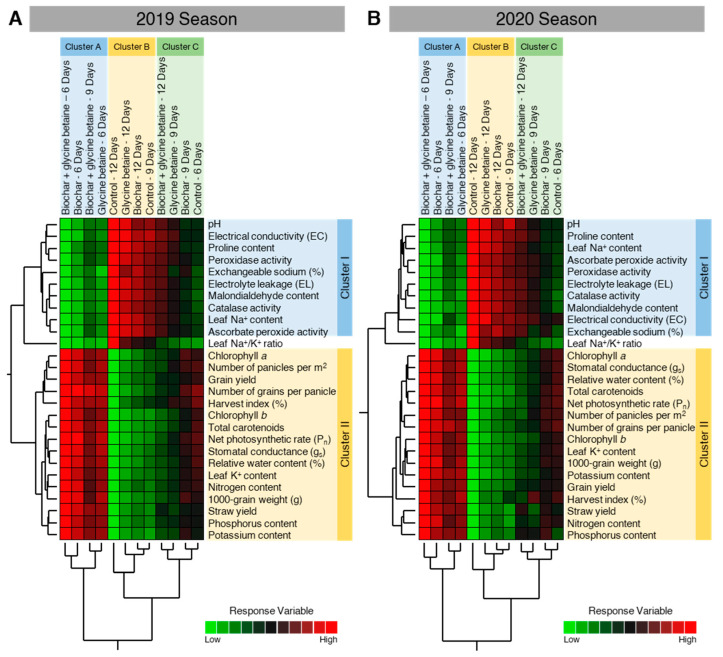
Two-way hierarchical cluster analysis (HCA) of individual response variables assessed in osmotic-stressed rice plants under three irrigation intervals (6, 9, and 12 days) during the 2019 and 2020 seasons. (**A**,**B**) Two-way HCA during the 2019 and 2020 seasons, respectively. Variations in the dependent variables among studied treatments are visualized as a heat map. Rows correspond to dependent variables, whereas columns correspond to different treatments. Low numerical values are green-colored, while high numerical values are colored red (see the scale at the right bottom corner of the heat map).

In agreement with our PCA findings, the HCA and its associated heatmap revealed the differences between treatments (Figure 11). Briefly, in both seasons, HCA-associated dendrogram among treatments showed that all treatments were clustered separately in three distinct clusters. Cluster A included biochar-treated, glycine betaine-treated, dual-treated (biochar + glycine betaine) at 6 days irrigation intervals, and dual-treated (biochar + glycine betaine) at 9 days irrigation intervals. Cluster B included non-treated control, glycine betaine-treated, biochar-treated at 12 days irrigation intervals, and non-treated control at 9 days irrigation intervals. Cluster C included dual-treated (biochar + glycine betaine) at 12 days irrigation intervals, glycine betaine-treated, biochar-treated at 9 days irrigation intervals, and non-treated control at 6 days irrigation intervals.

Moreover, the HCA-associated dendrogram among dependent variables showed that all tested variables were clearly clustered into two distinct clusters. Cluster ‘I’ included pH, electrical conductivity (EC), proline content, POX activity, exchangeable sodium, electrolyte leakage (EL), malondialdehyde content, CAT activity, leaf Na^+^ content, and APX activity, which were all higher in ‘Cluster B’ of the treatments. Cluster ‘II’ included chlorophyll *a*, number of panicles per m^2^, grain yield, number of grains per panicle, harvest index (%), chlorophyll *b*, total carotenoids, net photosynthetic rate (Pn), stomatal conductance (g_s_), relative water content, leaf K^+^ content, grain N content, 1000-grain weight, straw yield, grain P content, and grain K content, which were all higher in ‘Cluster A’ of the treatments. It is worth mentioning that the leaf Na^+^/K^+^ ratio was clustered separately in the middle of the dendrogram between Cluster ‘I’ and Cluster ‘II’ during 2019 (Figure 11A) and 2020 (Figure 11B).

## 3. Discussion

Rice is among the field crops susceptible to environmental stressors like water stress and soil salinity which restraint plant growth and development owing to the decline in the water holding capacity by plant roots due to the osmotic pressure alongside ionic toxicity [25,26]. In terms of botanical origin, rice is originally an aquatic plant that has a low tolerance to drought stress. It was found that biochemical and physiological modifications happen alongside augmented ROS [26]. Therefore, the enormous increment in the populace worldwide is forcing us strongly to focus on the use of our natural resources to save food security. Moreover, consumer’s demand for better nutritional and commercial quality foods has been raised. When Salinity and drought are combined, the soil fertility is reduced alongside plant growth, and productivity is declined, particularly in arid and semiarid zones such as Egypt [27]. It has been reported previously that rice plants shared common mechanisms for drought and salinity tolerance [12,13]. For example, quantitative trait loci (QTLs) have been reported to play a key role in drought, flood, and salt tolerance in mega rice varieties [13]. Recently, soil amendment such as biochar and foliar spraying like glycine betaine has found its way into maintaining agricultural sustainable development, which has a great potential for enhancing the performance of crop yield and mitigating the environmental stress [28,29]. It has been suggested, with strong evidence, that biochar amendment is a promising approach to mitigate soil contamination via immobilizing heavy metals [30], improving overall soil quality [31,32], enhancing water–fertilizer productivity [31], and decreasing soil salinity [33] in arid [33] and semi-arid areas worldwide [31,32]. Biochar could be produced from different sources (well-reviewed by Guo et al. [30]). Common biochar feedstocks extend to forest debris, crop residues, food processing waste, and manures [30,34]. However, the characteristics and functional capacity of different kinds of biochar differ extensively [30]. This might be due to the significant differences in organic and ash compositions of biomass materials [30,34]

### 3.1. Soil Physicochemical Properties

Soil salinity and water stress combined with high Na^+^ content has negatively affected soil structure, physicochemical properties, increased soil pH, and EC that limit root growth and plant development alongside constraint water uptake and nutrient imbalance owing to an antagonistic impact between Na^+^ and other elements such as Ca^2+^, K^+^, and other cations [25,26]. Application of biochar augmented K^+^, Ca^2+^, and Mg^2+^ contents in soil solution while declined Na^+^ content alongside soil pH and EC values resulting in decreased greatly exchangeable sodium percentage (ESP) due to biochar’s porous structure, high water holding capacity, large surface area, and negative surface charge that holds more nutrients under soil salinity and water stress conditions [35]. In the current investigation, the combined application of biochar with glycine betaine decreased greatly the exchangeable sodium percentage (ESP), soil pH, and EC values while augmented K^+^, Ca^2+^, and Mg^2+^ contents in soil solution that positively affected soil physicochemical properties causing better plant growth in rice under soil salinity and water stress conditions in both years of the study [18,36].

### 3.2. Ion Selectivity

The harmful impacts of soil salinity and water stress disturb rice plants through two faces; the first is owing to the high osmotic stress and the second is ion toxicity as a consequence of more accumulation of toxic ions like Na^+^ and Cl^−^ as found in the present work [37]. The application of biochar has a great potential to decrease Na^+^ uptake and increase K^+^ uptake. Biochar application increased cell formation and size in the root and allowed rice roots to absorb more K^+^ and hold Na^+^ ions away under salt stress avoiding ion toxicity, reducing the transport of Na^+^ into the xylem isolating Na^+^ into the vacuole [38,39]. Application of glycine betaine as foliar spraying improved seedling vigor, cell elongation, and growth, while enabling the plants with the developed transporter linked with the positive ion selectivity on the cell membrane and vacuole wall [40]. Application of glycine betaine reduced the harmful impact of Na^+^ ion toxicity and improved plant growth under harsh conditions compared to plants that were not treated with glycine betaine. In this context, the combined application of biochar [41] and glycine betaine could be a beneficial approach to address the growing problem of soil salinity and water stress.

### 3.3. Antioxidant Enzymatic Activity, Lipid Peroxidation, and Electrolyte Leakage

Water deficit causes a great increment in the enzymatic activity, i.e., CAT, APX, and POX, as well as lipid peroxidation (MDA) and electrolyte leakage (EL %) with a defense line versus the harmful impacts of water deficit and salt-affected soil in rice plants [42,43]. Augmenting the activity of antioxidant enzymes resulted in reducing the oxidative stress under drought and a salinity stress that diminishes ROS [44]. Application of soil amendment (biochar) enhanced CAT, APX, and POX activities as well as MDA and EL% that maintained membrane integrity and alleviated the harmful effects of water deficit and salt-affected soil [45]. Foliar spraying with glycine betaine to rice leaves alleviated salt-mediated oxidative stress, as significantly sustained by increasing the activity of antioxidant enzymes [46] that stimulate the plant development by supporting the protection versus oxidative stress through improving the antioxidant defense system; thus, rice develops tolerance mechanisms versus water deficit under salt-affected soil [47]. The exogenous addition of glycine betaine as foliar spraying proved its protective role against the accelerated oxidative stress and exposed positive effects on enhancing the enzymatic activity owing to its ability in increasing the electron transport chain alongside its beneficial effect as a promoter in protecting the plant cells from oxidative stress during its effect on osmoregulation, protein stabilization, and antioxidant equilibrium [48]. Thus, the combined application of biochar and glycine betaine caused more improvement in CAT, APX, and POX, as well as MDA and EL% than sole applications. Our findings are in harmony with the results of [49].

### 3.4. Physiological Traits

Combined water stress and salt-affected soil impede plant growth and development by detrimentally damaging osmolytes such as proline content, chlorophyll pigments (chlorophyll *a*, *b*, and carotenoids), and various physiological traits, such as the photosynthetic rate, stomatal conductance, and relative water content in rice plants due to deficient biosynthesis, as presented by Zhang et al. [49]. Likewise, the effects of different water regimes on agronomic characteristics, physiology, and grain quality, as well as photosynthesis and stomatic conductance of different elite quinoa genotypes under field conditions [50]. The enhancement of the osmolytes, chlorophyll pigments, and physiological processes could be ascribed to biochar application as a soil amendment in stimulating the meristematic activity, which results in augmenting cell division and enlargement [51]. Biochar application has the potential to holding water alongside increment nutrient absorption. Furthermore, IAA-producing bacteria improves soil quality such as the soil physicochemical properties [52]. It has been proven, indeed, that biochar can boost dry matter production (root and leaves) as a consequence of its positive impact on osmolytes, chlorophyll pigments, and physiological traits [28]. These results are consistent with earlier reports such as those shown by Zhang et al. [52]. It was proven that foliar application with glycine betaine is quickly taken up by leaf tissue and it accumulates principally in the cytosol. Therefore, it improves plant growth under harsh conditions owing to the stabilization of quaternary development of proteins and membrane integrity. Furthermore, foliar-applied with glycine betaine showed a substantial potential for improving the biosynthesis of chlorophyll pigments, such as chlorophyll *a*, chlorophyll *b*, and carotenoids as well as physiological measurements like photosynthetic rate, stomatal conductance, and relative water content under water stress and soil salinity in rice plants through nutrient content and hormone-like compounds [48]. Glycine betaine application as foliar spraying significantly reduced free proline content alongside improved chlorophyll pigments and physiological measurements in both seasons under harsh conditions due to osmoregulation and influencing hydraulic conductivity, similar data were reported by Irigoyen et al. [53]. Glycine betaine application has a direct contribution to cell respiration, photosynthesis, oxidative phosphorylation, protein polymerization, and other enzymatic reactions [54]. It was found that the combined application of biochar with glycine betaine had a more positive effect on osmolytes, chlorophyll pigments, and physiological measurements than a solo application under water deficit and soil salinity that induced mineralization, organic acids, and augmented plant nutrient availability. These findings are in agreement with those noted by Hasanuzzaman et al., 2014 and Huang et al., 2013 [55,56]. It is believed that the stimulatory effect of glycine betaine and biochar together on the permeability of the plasma membrane is responsible for the improvement of plant nutrition, through nutrient and water uptake.

### 3.5. Yield and Yield Components

In the present investigation, the decrement in the number of panicles/m^2^, 1000 grain weight (g), and the number of grains/panicles which negatively affecting grain yield, straw yield, and harvest index under water stress and soil salinity was ascribed to the inhibition in the absorption and transfer of the nutrition materials through the growth of grains and their filling periods. Furthermore, soil salinity can trigger harsh injury to the ovary, and thus may lead to a decrease in the yield [57]. In terms of yield and its components, significant differences were observed between different wheat genotypes growing under different water regimes in the Cerrado region in Brazil [58]. Moreover, the individual application of glycine betaine as foliar spraying or biochar application as soil amendment was more efficient to enhance rice yield components such as the number of panicles/m^2^, the number of grains/panicles, and 1000 grain weight (g) as a consequence of mitigating the harmful impact of water stress and soil salinity compared to untreated plants (control treatment) that can increase its panicle sterility under such a condition [59]. The high efficiency of glycine betaine could be owing to its potential to decline osmotic stress, decrement sodium uptake, increment potassium uptake maintaining healthy flag leaf, augmenting photosynthesis with high net assimilation rate, and translocation from sources to sink as well as starch accumulation in the chloroplast, decrease oxidative stress, delaying of senescence, and water status adjustment and increase the antioxidative capacity [60]. However, the high efficiency of biochar could be due to its potential to produce high seedling vigor with a free radical defense system [51]. In this respect, soil and foliar application are stated to increase the growth and yield of higher plants, especially under water stress and soil salinity [52]. All of the above-mentioned benefits were further developed by combined biochar and glycine betaine which showed high panicle fertility with low sterility combined with heavy panicle resulted in high yield under salt and water stressors.

### 3.6. Nutrient Status

Grain yield formation is the final product during plant growth and development that particularly relies on vigorous vegetative growth. Water stress and soil salinity reduced nutrient uptake from the soil as a consequence of overflowing Na^+^ ions. It was shown that nutrient uptake in terms of N, P, and K contents was negatively impacted by water stress and soil salinity [61]. The data might be ascribed to nutrient unavailability in the soil due to low soil moisture content and osmotic stress which declined the movement of cations and anions from soil to roots resulting in a decrease of transpiration rate and transport nutrients from roots to leaves resulting in decreasing physiological traits reflecting finally on the efficiency of yield production [62]. Biochar application enhanced soil nutrient cycling such as N, P, and K for plant absorption owing to biochar’s porous structure, large surface area, negative surface charge [51], increment the soil’s cation exchange capacity, and permit for the holding of nutrients resulting in increasing plant N, P and K concentrations under water deficit and soil salinity. Foliar-applied glycine betaine could mitigate water deficit and soil salinity through increasing leaf area [52], cell division, and improving chlorophyll pigments and physiological measurements [62]. Glycine betaine could also maintain soil moisture content and nutrient uptakes like N, P, and K and prevent Na influx which enhances leaf water content. It was proven that glycine betaine enhanced the transport of N, P, and K from leaves to grains to support grain formation [22]. The magnitude increment in N, P, and K contents in grains was more pronounced with the coupled application of biochar and glycine betaine.

## 4. Materials and Methods

### 4.1. Plant Materials and Experimental Design

Two field trials were performed during the two summer growing years of 2019 and 2020 at the Experimental Farm of El-Karada Water Requirements Research Station, Sakha, Kafr Elsheikh (North Delta), Egypt (Latitude: 31°6′ N/ Longitude: 30°56′ E). The preceding cultivated plant was wheat during the two years of the study. The objective was to report the dual effects of foliar spraying with glycine betaine and soil amendment with biochar under three irrigation intervals (every 6 (I_1_), 9 (I_2_), and 12 (I_3_) days) on soil properties, physiological parameters, yield-related traits, productivity well as grain quality of rice (*Oryza sativa* L., cv. Sakha 105) in salt-affected soil. The experiment was set up in a split-plot design with four replicates. The main plots were assigned to three irrigation intervals; irrigation every 6, 9, and 12 days after transplanting which is indicated as I_1_, I_2_, and I_3_ respectively. The subplots were allocated to four treatments namely, control (neither biochar nor glycine betaine applied and was done with distilled water); biochar as soil application (at the rate of 10 t ha^−1^); foliar application of glycine betaine (at the rate of 50 mM); and combined (biochar + glycine betaine). Main plots including irrigation intervals were tightly detached by trenches, 2 m width and 1 m depth. Treatment of glycine betaine had been sprayed thrice, at mid-tillering, panicle initiation, and full heading. Biochar added in this present investigation was prepared through slow pyrolysis of rice husk and corn stalk (1:1) at 350 °C under no oxygen conditions with an average residence time of 3 h [63]. Biochar was cut down in a stainless steel mill and sieved through a ~2-mm mesh to remove immense particles subsequent air drying and therefore machinery raked for leveling. During the ploughing process, biochar was allocated to every experimental unit and mixed thoroughly. Physico-chemical properties of used biochar are listed in Table S1.

Pre-germinated seeds (at the rate of 120 kg ha^−1^) were soaked in fresh water for 24 h and incubated for another 48 h to stimulate seedlings) and were broadcasted homogenously by hand in the nursery on 1 and 5 of May in the 2019 and 2020 seasons, respectively. Four seedlings per hill at thirty days old were transplanted in the permanent field’s experimental plots at a 20 cm × 20 cm distance between hills and rows in 67.5 m^2^ (13.5 m × 5.0 m) size plots. Weeds were controlled mechanically using hands and chemically using Saturn 50% (active ingredient Thiobencarb 800 g L^−1^) at the rate of 5 L ha^−1^ at 5 days post transplanting. Nitrogen was applied in the form of urea (46% N) on three equal doses during rice vegetative growth at the rate of 165 kg ha^−1^. The recommended phosphorous and Potassium fertilizers in the form of calcium superphosphate (15% P_2_O_5_) at a rate of 37 kg P_2_O_5_ ha^−1^ and potassium sulfate (48% K_2_O) at the rate of 50 kg K_2_O kg ha^−1^ were added during soil preparation. During the 2019 and 2020 seasons, representative soil samples were collected at the depth of 0–30 cm from the experimental site. The physical and chemical properties of the experimental soil were measured based on [64] and demonstrated in Table S2.

### 4.2. Soil Physicochemical Properties

At rice maturity from a 0–30 cm depth, representative soil samples were assembled via an auger. Soil samples were dehydrated in the open air and delivered across a 2-mm strainer for chemical traits assessment. The EC_e_ (dS m^−1^) was calculated in soil paste extract, whilst pH was estimated in 1:2.5 soil: sanitized water suspension, whereas pH was estimated by pH-meter (Genway, UK). The EC_e_ was computed by EC-meter (Genway, UK). The content (meq L^−1^) of Na^+^, K^+^, Ca^2+^, Mg^2+^ ions was estimated in soil paste extract via Atomic Absorption Spectrophotometer (AAS, Perkin Elmer 3300) with a detection limit of 100 ppb [65]. Exchangeable sodium percentage (ESP) was calculated using Equation (1) as described by Seilsepour et al. [66]:

ESP = 1.95 + 1.03 × SAR (R^2^ = 0.92) (1)

where SAR (Sodium adsorption ratio) was estimated using Equation (2) as suggested by Richards, 1954 [63]:

(2) $$\mathrm{SAR}=\left[{\mathrm{Na}}^{+}\right]/\sqrt{\frac{\left(\left[{\mathrm{Ca}}^{2}{}^{+}\right]+\left[{\mathrm{Mg}}^{2}{}^{+}\right]\right)}{2}}$$

where Na^+^, Ca^2+,^ and Mg^2+^ were computed in meq L^−1^.

### 4.3. Leaf Na^+^ and K^+^ Determination

At the anthesis stage, representative samples of five plants were randomly collected from each experimental unit to measure Na^+^ and K^+^ contents (mg kg^−1^ DW) via ultra-pure water the volume of the sample was brought to 50 mL in a volumetric flask. Based on Temminghoff and Houba [67], Na^+^ and K^+^ contents were estimated using AAS (Perkin Elmer 3300) with a detection limit of 100 ppb.

### 4.4. Physiological Measurements

Photosynthetic pigments (chlorophyll *a*, *b* and carotenoid; mg g^−1^ FW) were measured at the heading stage based on Peng, 1992 [68]. Ten representative leaves were rinsed to eliminate the impurities pre-extraction. Afterward, 2 g of the leaves were collected and homogenized in 80% acetone by the mortar and pestle. The extracts were centrifuged. The absorbance was computed at 663, 645, 470 nm by a spectrophotometer.

The net photosynthetic rate (P_n_) (μmol CO_2_ m^−2^ s^−1^) and stomatal conductance (g_s_) (mol m^−2^ s^−1^) were measured at the heading stage via a portable photosynthesis measurement system (Li-Cor, Lincoln, NE, USA) at 09:30–11:30 a.m. from flag leaves (fully expanded functional leaves). Air relative humidity ranging between 45–55%, ambient CO_2_ concentration was about 370 µmol CO_2_ mol^−1^ and a leaf temperature of 30 °C, respectively during collecting the data.

The relative water content (RWC) of rice leaves was assessed according to the method of Barrs and Weatherley [69]. Fresh rice leaves were scratch into minor pieces (1.5 cm length), and weighed fresh weight (FW, mg). Then, these leaves were put in distilled water for 4 h under low light to record turgid weight (TW, mg), and later oven-dried until constant weight at 80 °C for 24 h to measure dry weight (DW, mg). RWC was calculated using Equation (3):

(3) $$\mathrm{RWC}\;\left(\%\right)=\frac{\left(\mathrm{FW}-\mathrm{DW}\right)}{\left(\mathrm{TW}-\mathrm{DW}\right)}\times 100$$

Proline content was estimated based on the method of Bates et al. [70]. Roughly 300 mg of fully expanded functional leaves tissue was mixed in 10 mL of 3% (*w*/*v*) aqueous sulfosalicylic acid and sieved. To 2 mL of the filtrate was put on 2 mL of ninhydrin acid; later 2 mL of glacial acetic acid was put on the mixture, and the mixture was heated for 60 min. The absorbance readings of the toluene layer were read on a spectrophotometer at 520 nm. Proline content was computed as a standard curve. Proline content was described as µg g^−1^ fresh weight.

### 4.5. Assay of Antioxidant Enzymatic Activity and Lipid Peroxidation

The catalase (CAT; Unit mg^−1^ protein) activity was estimated by the suggested technique of Aebi, 1984 [71]. The test pipe included 100 µL of H_2_O_2_ (5.9 Mm) and 1000 µL buffer combined with the 100 µL of plant extract. The absorbance of samples was measured at 240 nm by spectrophotometer. For ascorbate peroxide (APX; Unit mg^−1^ protein) activity, the combination included 100 µL enzymes extracts, 100 µL ascorbate (7.5-mM), 100 µL H_2_O_2_ (300 mM), and 2.7 mL potassium buffer (25 mM), 2-mM EDTA having 7.0 pH. The content of APX was estimated at 290 nm wavelength by spectrophotometer. Peroxidase (POD; Unit mg^−1^ protein) activity was estimated by the technique of Vetter et al. [72]. The mixture of reactants including 100 µL extract enzyme + 2700 µL of 50 mM potassium buffers containing 0.25% (*v*/*v*) guaiacol and 100 mM H_2_O_2_. 100 µL was utilized for the measurement. The plant sample (0.5 g) was mixed with 5-mL potassium phosphate buffer (50 mM) with 7.0 pH under ice-cold circumstances and centrifuged at 15,000. The absorbance of the extract was measured at 470 nm for 2 min. Lipid peroxidation was assayed as Malondialdehyde (MDA) content in rice leaves according to Rao and Sresty [73], through with 5 mL of thiobarbituric (TBA) technique via MDA Detection Kit (A401; Sino Best Biological Co., Ltd., Beijing, China). After that, the absorbance for MDA was measured at 532 and 600 nm and stated as μmol g^−1^ FW.

### 4.6. Electrolyte Leakage (EL)

At the flowering stage, rice fully expanded functional leaves (flag leaves) were sampled and cut into small pieces, and placed into 20 mL of distilled water and the rate of leakage was read at 1-min intervals for 60 min via a conductivity meter (CM 100 conductivity meter, John E. Reid and Associates, Chicago, IL, USA). The leakage rate was measured as the slope of the line to the leaf dry weight by the method of Sullivan [74].

The electrolyte leakage (EL%) was calculated using Equation (4):

(4) $$\mathrm{EL}=\frac{\left(\mathrm{Initial}\;\mathrm{conductivity}\right)}{\left(\mathrm{Final}\;\mathrm{conductivity}\right)}\times 100$$

### 4.7. Plant Sample Harvest

At the maturity stage, panicles of five random hills from each experimental unit were counted then converted to the number of panicles/m^2^. Panicles number was measured from every hill, each panicle was hand-threshed and the unfilled panicles were separated from filled panicles using a blower. Ten panicles were randomly collected from each experimental unit to estimate the number of grains/panicles and 1000 grain weight (g). The biological yield (both grain and straw yield t ha^−1^) was measured from a 6-m^2^ area in each experimental unit except the outer border, and the standard grain moisture content of 14% was added to yield computation, as expressed by Yoshida, 1981 [75]. Harvest index (HI; %) was computed as the ratio between grain and biological yields and expressed as %. HI (%) was calculated using Equation (5):

(5) $$\mathrm{Harvest}\;\mathrm{index}=\frac{\mathrm{Grain}\;\mathrm{yield}\;\left({\mathrm{kg}\;\mathrm{ha}}^{-1}\right)}{\mathrm{Biological}\;\mathrm{yield}\;\left({\mathrm{kg}\;\mathrm{ha}}^{-1}\right)}\times 100$$

### 4.8. Nutrient Analysis

At harvest, five panicles were taken randomly from the inner of the experimental unit. Thirty kernels were taken from each plot, washed with distilled water, and oven-dried for 48 h at 70 °C to estimate grain N, P, and K contents (g kg^−1^). The dried samples were ground with a stainless-steel grinder and digested with HNO_3_ (70% *v*/*v*):H_2_O_2_ (30% *v*/*v*) solution (2:1). P content was calorimetrically estimated based on the method of Sparks et al. [76]. The K content was assessed via AAS (Perkin Elmer 3300) with a detection limit of 100 ppb [76]. Another 1-g powder aliquot was digested with concentrated sulfuric acid to assess the N content via the Kjeldahl method [77].

### 4.9. Statistical Analysis

In the current study, a full factorial experiment design (strip-plot) was used as an experimental layout in all experiments. Our experimental design consists of two factors: (1) three levels of irrigation intervals (6, 9, and 12 days) as the whole plots and (2) four treatments (control, biochar, glycine betaine, biochar + glycine betaine) as strips. All experiments were repeated twice in two different seasons with at least three biological replicates for each treatment. The analysis of variance (ANOVA) was used to test the significant differences among treatments. Based on the strip-plot ANOVA, three *p*-values were mentioned; (1) *p*-value for the whole plots (reported as *P*_IR_) to compare between different irrigation intervals, (2) *p*-value for the strips (reported as *P*_TR_) to compare between treatments, and (3) *p*-value of the interaction between irrigation intervals and treatments (mentioned as *P*_IR×TR_). Moreover, Tukey’s honestly significant difference (HSD) test was used for post-hoc analysis (*p* < 0.05). ANOVA and Tukey’s test were carried out using JMP Data analysis software-Version 15 (SAS Institute Inc., Cary, NC, USA). Moreover, the data matrix of all individual response variables was used to perform the principal component analysis (PCA) and its associated scatter and loading plots. Finally, the standardized means of all individual response variables were used for two-way hierarchical cluster analysis (HCA). Similarities and variations between treatments are presented as a heat map.

## 5. Conclusions

Our findings suggest that incorporated biochar-based soil amendment and exogenous glycine betaine foliar application can be an effective, sustainable, eco-friendly strategy to improve the resilience of rice plants growing in salt-affected soils especially in arid and semiarid regions. Dual application of biochar + glycine betaine significantly enhanced the growth, physiology, productivity, grain quality, and osmotic stress tolerance of rice plants, as well as soil properties and nutrient uptake during two successive seasons under field conditions. Our findings show that the beneficial role of biochar and glycine betaine might be due to the activation of the enzymatic antioxidant defense machinery to maintain reactive oxygen species (ROS) homeostasis within stressed plants. However, further studies are required to deeply investigate the long-term effect(s) of biochar and glycine betaine on both plant and soil ecosystems.

## Data Availability

The data that supports the findings of this study are contained within the article or supplementary material and available from the corresponding author upon reasonable request.

## Supplementary Materials

The following are available online at https://www.mdpi.com/article/10.3390/plants10091930/s1, Table S1: Soil physicochemical attributes used in the two successive seasons 2019 and 2020; Table S2: Physico-chemical properties of biochar (rice husk and corn stalk [1:1]) used in the two successive seasons 2019 and 2020.
