# Supplementary material for: Incorporated Biochar-Based Soil Amendment and Exogenous Glycine Betaine Foliar Application Ameliorate Rice (Oryza sativa L.) Tolerance and Resilience to Osmotic Stress
Source: Plants (Basel). 2021 Sep 16;10(9):1930. doi: 10.3390/plants10091930 (PMC8471834; doi:10.3390/plants10091930)
Supplement: Supplementary file 1 [file plants-10-01930-s001.zip › Hafez et al., RICE - for Plants - Supplementary materials v1.pdf]

plants

MDPI

Supplementary materials

Incorporated biochar-based soil amendment and exogenous glycine betaine foliar application ameliorate rice (*Oryza Sativa* L.) tolerance and resilience to osmotic stress

Emad M. Hafez [1], Salah M. Gowayed [2], Yasser Nehela [3,4,*], Raghda M. Sakran [5], Asmaa M. S. Rady [6], Abdelmoniem Awadalla [7], Alaa El-Dein Omara [8], Bassam F. Alowaiesh [9]

[1] Department of Agronomy, Faculty of Agriculture, Kafrelsheikh University, Kafr El-Sheikh 33516, Egypt; emadhafez2012@agr.kfs.edu.eg
[2] Department of Botany, Faculty of Agriculture, Suez Canal University, Ismailia 41522, Egypt; salahgowed@yahoo.com
[3] Department of Agricultural Botany, Faculty of Agriculture, Tanta University, Tanta 31527, Egypt; yasser.nehela@ufl.edu
[4] Citrus Research and Education Center, Department of Plant Pathology, University of Florida, 700 Experiment Station Rd., Lake Alfred, FL 33850, USA
[5] Rice Research Department, Field Crops Research Institute, Agricultural Research Center, Egypt; raghdasakran@yahoo.com
[6] Crop Science Department, Faculty of Agriculture (EL-Shatby), Alexandria University, Alexandria 21545, Egypt; asmaa.mohamed@alexu.edu.eg
[7] Department of Agronomy, Faculty of Agriculture and Natural Resources, Aswan University, Aswan 81528, Egypt; abdelmonemomr@yahoo.com
[8] Department of Microbiology, Soils, Water and Environment Research Institute, Agricultural Research Center, Giza 12112, Egypt; ala.omara@yahoo.com
[9] Olive Research Center, Jouf University, Sakaka, Aljouf 72341, Saudi Arabia; olive@ju.edu.sa
* Correspondence: yasser.nehela@ufl.edu

plants

Table S1. Physico-chemical properties of biochar (rice husk and corn stalk [1:1]) used in the two successive seasons 20 19and 2020.

Attribute	Biochar	Unit
pH (1:5 biochar:water extract)	7.60 ± 0.02	-
EC (1:5 biochar:water extract)	0.70 ± 0.01	dS m^{-1}
$CaCO_3$	1.4 ± 0.03	%
bulk density	0.20 ± 0.03	g cm^{-3}
specific surface area	37.0 ± 2.13	m^2 g^{-1}
water holding capacity	350 ± 12.23	%
moisture content	11.4 ± 1.09	%
Nitrogen	25.21 ± 2.91	mg kg^{-1}
Phosphorus	7.45 ± 0.83	mg kg^{-1}
Potassium	13.21 ± 1.42	mg kg^{-1}
Sulfur	90.25± 5.35	mg kg^{-1}
Sodium	8.36± 0.89	mg kg^{-1}
Chloride	35.47± 2.02	mg kg^{-1}

plants

MDPI

Table S2. Soil physicochemical attributes used in the two successive seasons 20 19and 2020.

				Cations (meq L^{-1})				Anions (meq L^{-1})		
Season	O.M (%)	EC (dS m^{-1})	pH	Na^{+}	K^{+}	Mg^{++}	Ca^{++}	Cl^{-}	HCO_3^{-}	SO_4^{--}
**2019**	1.38	5.84	8.11	27.85	0.37	6.45	9.78	24.65	4.32	15.22
**2020**	1.42	5.73	8.24	23.74	0.36	5.75	7.55	18.47	3.45	11.25

O.M = organic matter, EC= electrical conductivity

plants
